# F2R and MXRA5: the metabolic obesity-derived biomarkers for immunosuppression and poor survival in triple-negative breast cancer

**DOI:** 10.1007/s12672-026-04963-9

**Published:** 2026-04-12

**Authors:** Xia Li, Zhi Wang, Xiao Huo, Yi Luo

**Affiliations:** 1 Department of Oncology, Key Laboratory of Immunity, Inflammation & Cancer (Chongqing Municipal Health Commission), The First Affiliated Hospital of Chongqing Medical University, Chongqing, 400016 China; 2 Cancer Center, Union Hospital, Tongji Medical College, Huazhong University of Science and Technology, Wuhan, 430022 China; 3 Department of gastroenterology, The First Affiliated Hospital of Chongqing Medical University, Chongqing, 400016 China

**Keywords:** Breast cancer, Obesity, YAP/TAZ pathway, Biomarkers, Immunosuppression

## Abstract

**Objectives:**

The YAP/TAZ pathway is implicated in both obesity and breast cancer (BRCA), but the specific effector genes common to both diseases are unknown. We aimed to identify these shared YAP/TAZ effectors.

**Methods:**

We integrated transcriptomic data from BRCA (TCGA, GSE42568) and obesity (GSE25401, GSE151839) datasets. YAP/TAZ-related genes were identified through differential expression analysis, weighted gene co-expression network analysis (WGCNA), and machine learning algorithms. The tumor immune microenvironment was profiled using xCell, ESTIMATE, IPS and TIDE algorithms. The prognostic value was assessed in an independent cohort (GSE25065). Experimental validation was performed using clinical specimens via RT-qPCR.

**Results:**

F2R and MXRA5 were identified as core YAP/TAZ-effector genes associated with obesity and BRCA. F2R was upregulated in obese adipose tissue, and both genes exhibited transcriptional dysregulation in BRCA cohorts, though not all differences reached statistical significance between tumor and adjacent normal tissues. Comprehensive immune profiling showed that high expression of F2R/MXRA5 was associated with an immunosuppressive microenvironment, exhibiting reduced infiltration of dendritic cells and macrophages, elevated TIDE scores, and decreased tumor purity. Critically, high expression of either gene predicted significantly poorer overall survival in patients with triple-negative breast cancer (TNBC). Furthermore, computational analysis revealed a positive correlation between F2R/MXRA5 expression and predicted resistance to the HDAC inhibitor Vorinostat, suggesting a potential association with epigenetic therapy resistance.

**Conclusion:**

F2R and MXRA5 are novel biomarkers linking obesity to BRCA immunosuppression and poor outcomes, offering potential for risk stratification and combination therapy.

**Supplementary Information:**

The online version contains supplementary material available at 10.1007/s12672-026-04963-9.

## Introduction

Breast cancer (BRCA) and obesity (OB), two pressing health issues worldwide, are closely linked through biological and population-level factors. Breast cancer remains the most prevalent malignancy among women worldwide, consistently ranking first in incidence among all female cancers. Globally, both the annual number of newly diagnosed cases and mortality rates continue to occupy prominent positions in women’s cancer statistics [1]. Obesity, defined as abnormal or excessive fat accumulation, contributes to metabolic dysregulation and chronic inflammation, significantly increasing the risk of various cancers, including BRCA [2, 3]. Epidemiology studies reveal that obesity-related metabolic syndrome elevates breast cancer incidence, particularly in ER-positive patients [4] and reduces cancer-specific survival across all subtypes [5], highlighting the urgent need to identify shared molecular mechanisms.

The YAP/TAZ pathway, a key effector of the Hippo signaling cascade, regulates cell proliferation, tissue homeostasis, and organ size [6]. Dysregulation of YAP/TAZ is implicated in tumorigenesis and metabolic disorders [7]. In BRCA, YAP/TAZ activation promotes tumor cell survival, migration, and chemoresistance [8]. In obesity, YAP/TAZ drives adipocyte expansion and inflammation, exacerbating metabolic dysfunction [9]. A The study reveals that dysfunctional adipocytes drive tumor progression by activating the YAP/TAZ signaling pathway, a mechanism identified in both cancer - associated adipocytes and those deficient in BECN1 [10]. Despite these findings, there remains a significant research gap in the systematic identification of core YAP/TAZ effector genes consistently dysregulated in both obesity and breast cancer, and in elucidating their effects on the tumor immune microenvironment and clinical prognosis. Most existing studies have examined this pathway in isolation, either in cancer or metabolism [11, 12], lacking the recognition of shared transcriptional regulators that connect these two conditions. Furthermore, whether such shared genes could serve as risk-stratification biomarkers for obesity-associated breast cancer or predict responses to epigenetic or immunotherapies remains largely unexplored.

To investigate the YAP/TAZ pathway in obesity-associated breast cancer progression, we integrated BRCA and OB transcriptomes through multi-cohort analysis. Differential expression profiling, WGCNA, and machine learning identified YAP/TAZ-regulated biomarkers, validated via cross-cohort consistency, functional enrichment. Artificial neural network models assessed diagnostic potential, while drug sensitivity profiling and immune microenvironment analysis explored therapeutic implications. Additionally, we assessed the immunosuppressive tumor microenvironment and potential for immune escape through TIDE, IPS, and tumor purity analyses. Finally, we validated the expression of the biomarkers using clinical specimens and assessed their impact on breast cancer survival utilizing an external dataset. This study bridges computational predictions to clinically actionable targets, advancing mechanistic insights and precision strategies for obesity-driven BRCA.

## Materials and methods

### Data source

Publicly accessible transcriptomic datasets were utilized in this study. Breast cancer RNA-seq profiles were obtained from The Cancer Genome Atlas (TCGA, accessed 12 October 2024), including 1,113 tumor and 113 normal tissue samples that constituted training cohort 1 [13]. An independent GEO dataset (GSE42568, platform GPL570) containing 104 breast cancer and 17 control samples served as validation cohort 1 [14]. This dataset employed the standard Affymetrix preprocessing workflow, comprising Robust Multi-array Average (RMA) background correction, quantile normalization, and median aggregation.

Moreover, the transcriptomic datasets associated with OB were also obtained from the GEO database. The GSE25401 dataset, employing the GPL6244 platform, contained abdominal subcutaneous adipose tissue (aSAT) samples from 30 OB patients compared to 26 control individuals and was used as training cohort 2 [15]. The dataset GSE151839, on the GPL570 platform, encompassed fat tissue samples from 10 OB patients and 10 controls, serving as validation cohort 2 [16]. The processing workflow for the GSE25401 dataset was as follows: abdominal subcutaneous adipose biopsies from 56 women with varying Body Mass Index (BMI) were analyzed. Complementary RNA was hybridized to Affymetrix GeneChip Human Gene 1.0 ST Arrays. Gene expression analysis was performed using Affymetrix GeneChip Operating Software (v 1.4), with all samples normalized to a target signal of 100 to enable cross-sample transcript comparison. For the GSE151839 dataset, expression measures were obtained using the GCRMA algorithm. A batch effect corresponding to the hybridization date was detected by Principal Component Analysis (PCA) and adjusted using the ComBat function from the SVA package. A unified normalization strategy was applied, including dataset-specific log2(x + 1) transformation (or inverse transformation when necessary), gene-level probe deduplication by retaining the probe with the highest mean expression, filtering for protein-coding genes, and removal of missing values to ensure cross-dataset comparability and consistency. Additionally, a comprehensive query was performed in the MSigDB utilizing the search term “YAP/TAZ”, leading to the identification of a functionally relevant gene set named ORDENONSI_YAP_CONSERVED_SIGNATURE, comprising a total of 57 YT-RGs (Supplementary Table 1).

It should be noted that this study utilized abdominal subcutaneous adipose tissue (SAT) as the systemic model for obesity, primarily based on two considerations: first, SAT is a classical model for studying obesity-related metabolism and inflammation, and its transcriptome can effectively reflect the systemic state of obesity [17]; second, publicly available transcriptomic datasets of mammary adipose tissue with sufficient sample size are currently scarce. Therefore, by using SAT data to screen for YAP/TAZ downstream genes shared between systemic obesity and breast cancer, this study aimed to identify common molecular features driven by systemic obesity that may affect distal organs (such as the mammary gland), which is fully aligned with our research objective of focusing on the cross-disease core molecular association of “obesity-YAP/TAZ-breast cancer”.

### Selection of key module genes

To construct unbiased co-expression networks, the full gene expression matrices from TCGA-BRCA and GSE25401 were used as input for WGCNA, performed with the WGCNA package (v1.7.1) [18]. Prior to network construction, we calculated YAP/TAZ-related genes (YT-RGs) enrichment scores using ssGSEA from the GSVA package (v1.50.0) [19], which were compared between disease and control groups (*p* < 0.05) and then used as phenotypic traits. The analyses began with a clustering assessment of all samples based on gene expression data to identify potential outliers or anomalies. To establish scale-free networks, the ideal soft-thresholds (power) were determined by setting the scale-free fit indexes (R^2^) to 0.85 for BRCA and 0.80 for OB, while ensuring that the mean connectivity approached 0. Dendrograms of gene similarity were plotted (the minimum number of genes per module (minModuleSize) = 200, deepSplit = 1, and mergeCutHeight = 0.4), and correlations between modules and YT-RGs scores were calculated. Corresponding heatmaps were then generated to visualize these correlations. The modules exhibiting the highest correlation with YT-RGs scores were selected as key module 1 and key module 2 (|cor| > 0.30, *p* < 0.05) in training cohorts 1 and 2, respectively. Lastly, genes within key module 1 were designated as key module genes 1, and genes within key module 2 were classified as key module genes 2.

### Identification of intersecting genes and candidate genes

Differentially expressed genes (DEGs) between breast cancer and control samples in training cohort 1 were identified using DESeq2 (v1.42.0) [20], with thresholds set at |log2 fold change| > 0.5 and *p* < 0.05, yielding DEGs1. Similarly, DEGs in the obesity dataset were detected using the limma package (v3.44.3) [21] under the same criteria, designated as DEGs2. To control the false discovery rate (FDR) in high-throughput analyses, the Benjamini-Hochberg (BH) method was applied. While candidate genes were initially screened based on *P* < 0.05, the final biomarkers were validated to ensure an adjusted *P* < 0.05 to guarantee the reliability of the results. The volcano plots were generated to visualize DEGs1 and DEGs2 utilizing the ggplot2 (v 3.4.4) package [22], respectively, with the top 10 up- and down-regulated genes labeled by their p values. Concurrently, these top 10 up- and down-regulated DEGs1 and DEGs2 were further visualized through heatmaps employing ComplexHeatmap (v 2.16.0) package [22], respectively, sorted by p values from low to high.

Subsequently, using the ggvenn (v 0.1.9) package [23], the intersecting genes that were up-regulated across the DEGs1 and DEGs2, as well as those down-regulated across the DEGs1 and DEGs2, were identified, resulting in the formation of intersecting gene set 1 and intersecting gene set 2, respectively. Following this, by taking the union of these 2 intersecting gene sets, the intersecting genes were obtained for this study. Further analysis involved taking the intersection of intersecting genes, key module genes 1, and key module genes 2 to identify candidate genes, which were visualized using ggvenn (v 0.1.9) package.

### Enrichment analysis of candidate genes

Subsequently, Functional annotation of candidate genes was performed using Gene Ontology (GO) and KEGG pathway analyses via clusterProfiler (v4.10.0) [24], with significance set at *p* < 0.05. For each GO category and KEGG, the 10 most significantly enriched pathways (sorted by ascending p value) were selected for visualization. Protein–protein interactions among candidate genes were retrieved from STRING (confidence score ≥ 0.15) and visualized in Cytoscape (v3.10.2) [25] to construct the PPI network.

### Recognition of biomarkers

Candidate genes were subjected to LASSO regression (glmnet v4.1.4) [26] within each training cohort. Ten-fold cross-validation (nfolds = 10) was used to determine the optimal λ value. The default λ sequence generated by the algorithm (approximately 100 logarithmically spaced values, starting from a sufficiently large λ that shrinks all coefficients to zero) was applied. The optimal λ was chosen to minimize cross-validation error, and genes retained under this λ were considered candidate biomarkers for breast cancer or obesity. Subsequent analyses explored the expression trends of these candidate biomarkers in their respective cohorts. With a view to enhance reproducibility and minimize random errors and biases, validation cohorts 1 and 2 were also used to analyze expression trends, respectively. Box plots illustrated the expression trends of these candidate biomarkers.

In summary, genes that exhibited significant expression differences and consistent trends in both training cohort 1 and validation cohort 1 (*p* < 0.05) were defined as biomarkers for BRCA. Similarly, genes that showed significant expression differences and consistent trends in both training cohort 2 and validation cohort 2 (*p* < 0.05) were identified as biomarkers for OB. The intersection of biomarkers for BRCA and biomarkers for OB was then determined by applying VennDiagram (v 1.7.3) package [27] to identify biomarkers related to the YAP/TAZ pathway in both BRCA and OB. Based on the identified biomarkers, the ROC curves were generated using the pROC (v 1.18.0) package [28] to assess their predictive performance for BRCA and OB. The AUC values were calculated to quantitatively evaluate the biomarkers in training cohort 1 and validation cohort 1 (AUC > 0.7). Similarly, the predictive performance of each biomarker was assessed in training cohort 2 and validation cohort 2 using the same method (AUC > 0.7).

### Construction of the artificial neural network (ANN) models

Predictive artificial neural networks (ANN) were constructed with neuralnet (v1.44.2) [29] using biomarker expression values. The error function was defined as sum of squared errors (err.fct = “sse”), and linear.output = FALSE was applied to enable a sigmoid activation function suitable for binary classification tasks. Model performance was evaluated by ROC analysis (pROC v1.18.0); AUC > 0.7 was required in both training and validation sets.

### Functional and annotation analysis

To explore the functional implications of each biomarker, we performed gene set enrichment analysis (GSEA) using the clusterProfiler package (v4.10.0). For each biomarker, Spearman correlation coefficients with all genes were calculated via the psych package (v2.2.5) [30], and genes were ranked accordingly. The MSigDB C2 curated gene set (c2.cp.kegg.v2023.1.Hs.symbols.gmt) served as the reference database, with thresholds set at |NES| > 1 and adjusted *p* < 0.05. Finally, the top 5 pathways from the enrichment results for each biomarker were visualized in each cohort, ordered by adj.p values from low to high.

### Immune infiltration analysis

We estimated the abundance of 64 immune cell subsets per sample using the xCell algorithm (v1.1.0) [31]. Immune and stromal cell infiltration was quantified using the xCellAnalysis function to estimate the relative abundance of 64 cell types based on the expression matrix. Following sample grouping, differential infiltration patterns were analyzed via heatmaps and box plots with Wilcoxon rank-sum test, with *p* < 0.05 considered significant .Furthermore, Spearman correlation analyses were constructed through psych (v 2.2.5) package to investigate potential correlations between differential immune infiltrating cells with significant differences, as well as between differential immune infiltrating cells and biomarkers in these 2 cohorts (|cor| > 0.30, *p* < 0.05), respectively. These correlation relationships were then visualized using heatmaps. In addition, CIBERSORT was performed using the LM22 signature matrix to quantify the relative proportions of 22 immune cell types. Only samples with CIBERSORT output *p* < 0.05 were retained for subsequent analysis.

### Immunotherapy response assessment and tumor purity analysis

To explore whether F2R and MXRA5 influence immunotherapy efficacy, we retrieved IPS and TIDE scores from The Cancer Immunome Atlas (TCIA) [32]. Samples were split into high and low biomarker expression groups using median cut-offs and merged with IPS subtypes (ips_ctla4_neg_pd1_neg, ips_ctla4_neg_pd1_pos). TIDE scores were calculated to estimate T-cell dysfunction and immune exclusion. Differences in IPS and TIDE between groups were tested with the Wilcoxon rank-sum test and displayed as split-violin plots with boxed medians (significance: *P* < 0.05, *P* < 0.01). Microenvironment composition was quantified by ESTIMATE, yielding Stromal, Immune, ESTIMATE scores and Tumor Purity; comparisons were visualized with overlaid violin-box plots.

### Construction of regulatory network

miRNAs targeting each biomarker were predicted with multiMiR (v1.16.0) [33]. Transcription factors (TFs) were obtained from hTFtarget, retaining the five highest-scoring TFs per gene. Interactions were merged into a TF–mRNA–miRNA network and visualized in Cytoscape (v3.10.2).

### Chemotherapeutic drug sensitivity and drug prediction analyses

Chemosensitivity was estimated with pRRophetic (v0.5.1) [34] using GDSC-derived IC50 values for training-cohort-1 samples. The five drugs with lowest median IC50 were selected; correlations between biomarker expression and log-IC50 were computed with psych (|r| > 0.30, *p* < 0.05).

To further explore potential therapeutic drugs for OB, the DSigDB (https://dsigdb.tanlab.org/DSigDBv1.0/) was consulted to pinpoint drugs potentially associated with the biomarkers, and the biomarkers-drugs network was then mapped by Cytoscape (v 3.10.2) software.

### Expression analysis of biomarkers

This study was conducted following the ethical guidelines of the Declaration of Helsinki. The protocol was reviewed and approved by the Institutional Ethics Review Board of The First Affiliated Hospital of Chongqing Medical University (Approval No: 2025-439-01). Written informed consent was obtained from all participants. For this study, we analyzed paired tumor and adjacent non-tumor tissues from five patients with breast cancer. We also analyzed mammary adipose tissues from four normal-weight and four obese patients benign breast disease. The expression of biomarkers was further verified through reverse transcription-polymerase chain reaction (RT-qPCR). RNA was extracted with TRIzol, reverse-transcribed using PrimeScript RT, and quantified by SYBR Green qPCR on a StepOnePlus instrument. Each 20-µL reaction contained 0.4 µM primers (F2R: F-5′-CCCGCAGGCCAGAATCAAAA-3′, R-5′-CCTGAGAAGAAATGACCGGGG-3′; MXRA5: F-5′-TCCCAAGGACAGGTATCCGA-3′, R-5′-GACGTAGCAGGCACAAGGAT-3′). Thermal cycling began with 95 °C for 10 min, followed by 40 cycles of 95 °C 15 s and 60 °C 1 min; melt-curve analysis verified specificity [35]. Relative expression was calculated via the 2 − ΔΔCt method and plotted in GraphPad Prism v8.0.

### Validation of prognostic efficacy in an external cohort

We downloaded the GSE25065 dataset (198 breast cancer cases) and excluded samples with < 1 year follow-up. Optimal gene-expression cut-offs were identified with survminer::surv_cutpoint [36], allowing stratification into high- and low-expression cohorts for survival comparison across subtypes.

### Statistical analysis

All computations were performed in R v4.3.1. Two-group comparisons were carried out using the Wilcoxon rank-sum test, with *p* < 0.05 considered statistically significant.

## Results

### Recognition of 410 key module genes 1 and 588 key module genes 2

**Fig. 1:**
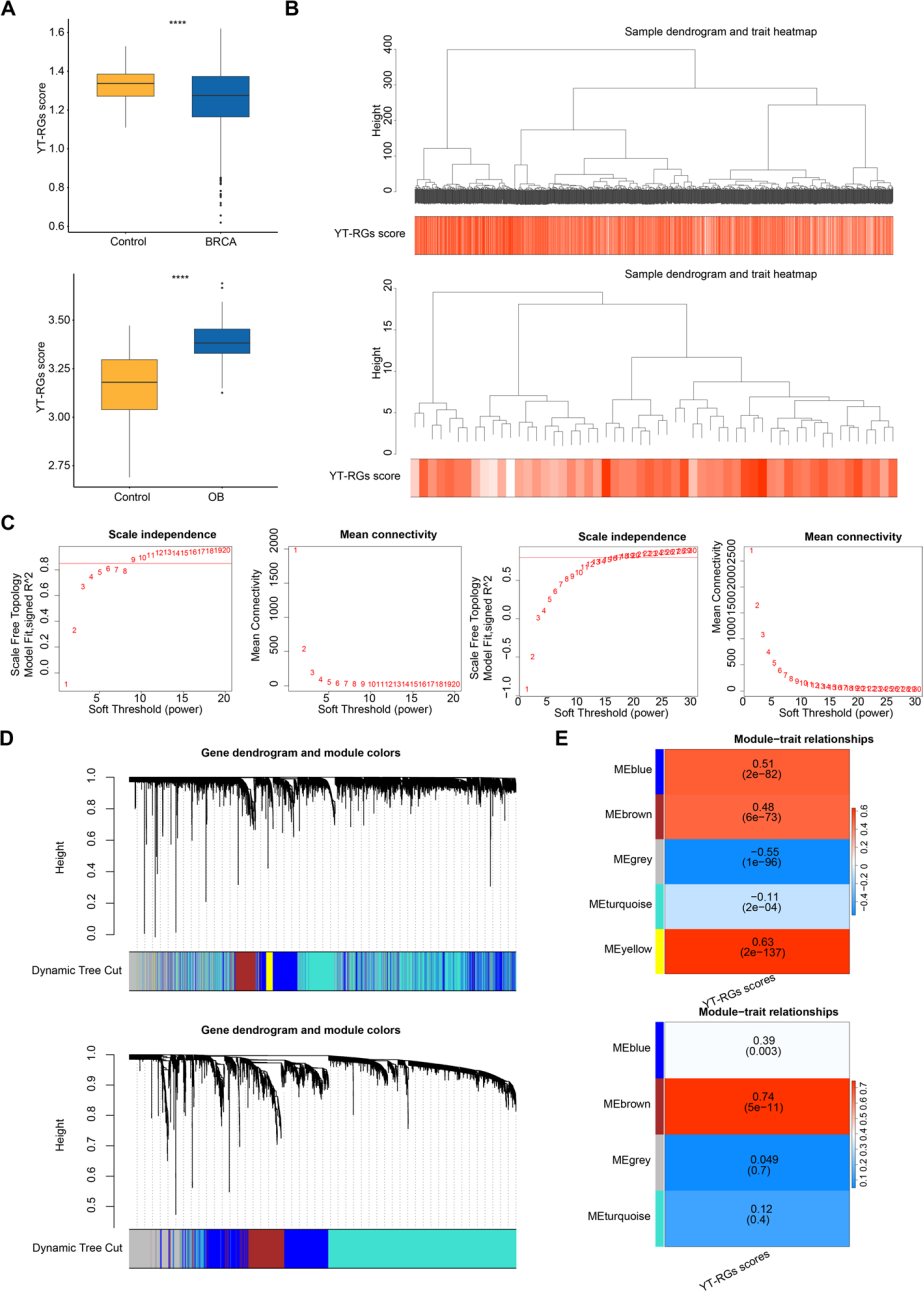
Integrated analysis of YAP/TAZ-related genes and WGCNA modules in breast cancer and obesity. **A** YTRGs(YAP-TAZ pathway related genes)scores in BRCA patient samples and control samples and OB patient samples and control samples; **B** WGCNA analysis and hierarchical clustering of all samples in Training Set 1and Training Set 2; **C** Scale-free topology fit and soft threshold power distribution for Training Set 1 and Training Set 2; **D** Module clustering dendrogram for Training Set 1and Training Set 2; **E** Heatmap of module-trait correlations in Training Set 1and Training Set 2

In cohort 1, YAP/TAZ-related gene scores were markedly lower in breast-cancer tissues than in normal controls (*p* < 0.0001), whereas obesity samples exhibited elevated scores (*p* < 0.0001) (Fig. 1A).

Subsequently, YT-RGs scores were utilized as traits for WGCNA, aimed at identifying key module genes associated with YT-RGs scores in BRCA and OB, respectively. Specifically, clustering of the samples revealed no outlier in both training cohorts 1 and 2 (Fig. 1B). The optimal soft-thresholds (power) were determined to be 9 for BRCA and 20 for OB, corresponding to the scale-free topology fit indexes (R²) of 0.85 and 0.80, respectively, with the mean connectivity approaching zero in both instances (Fig. 1C). By setting the minModuleSize to 200, deepSplit to 1, and mergeCutHeight to 0.4, a total of 5 modules were identified in BRCA and 4 modules in OB, including a grey module that could not be classified (Fig. 1D). The MEyellow module in BRCA demonstrated the strongest positive correlation with YT-RGs scores (cor = 0.63, *p* < 0.001) (Fig. 1E), and was designated as the key module 1, comprising 410 genes selected as the key module genes 1. Meanwhile, the MEbrown module in OB exhibited the strongest positive correlation with YT-RGs scores (cor = 0.74, *p* < 0.001) (Fig. 1E), and was designated as key module 2, including 588 genes selected as key module genes 2.

### Identification of 178 intersecting genes and 27 candidate genes

**Fig. 2:**
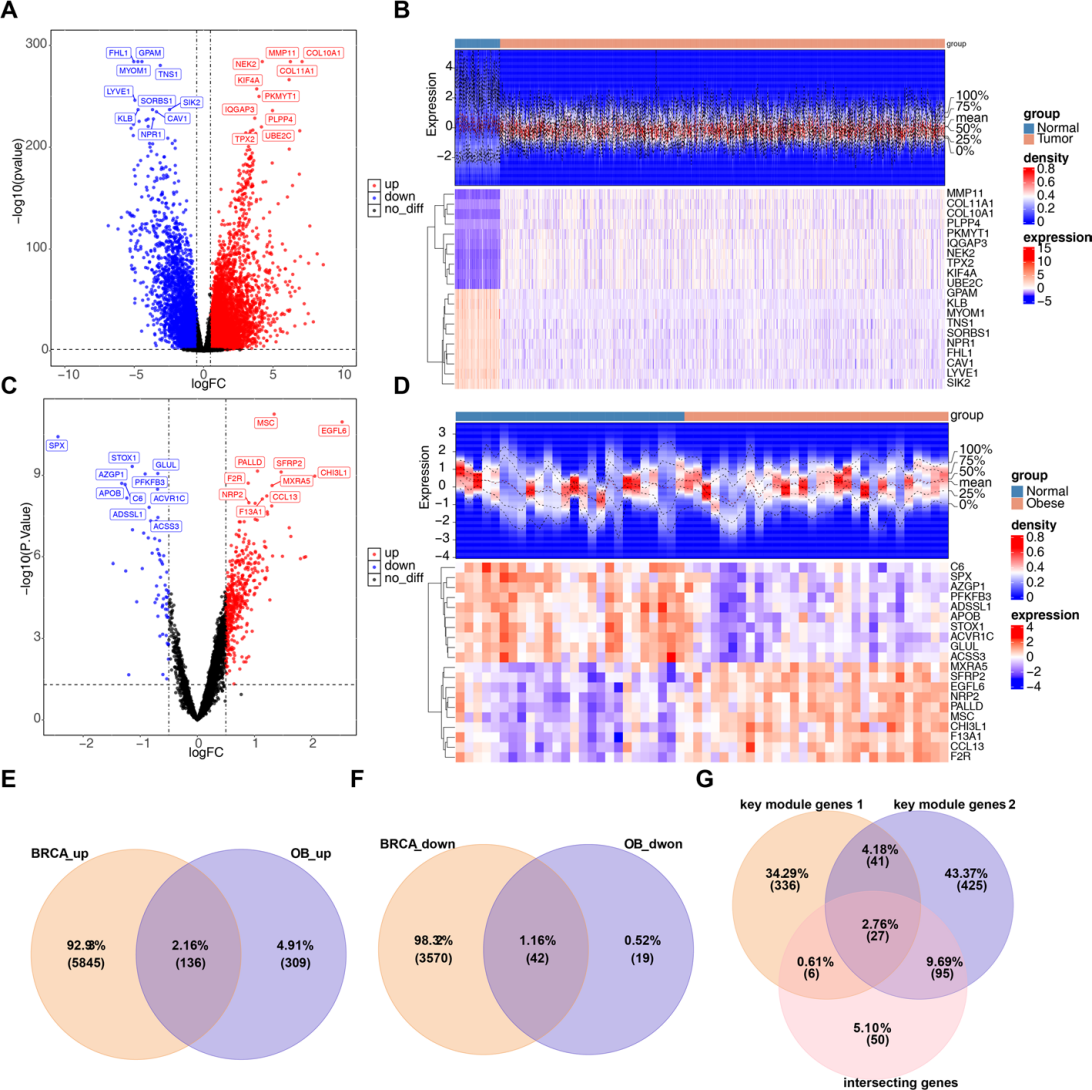
Differential gene expression and identification of candidate genes. **A** Volcano plot of DEGs1 (Differentially Expressed Genes, Group 1); **B** DEGs1 Heatmap; **C** Volcano plot of DEGs2; **D** DEGs2 Heatmap; **E** Common upregulated DEGs across Training Set 1 and Training Set 2; **F** Common downregulated DEGs across Training Set 1 and Training Set 2; **G** Venn diagram of candidate genes and YTRMGs1 (YAP-TAZ pathway related module genes1)/YTRMGs2

Following the differential expression analysis conducted in the training cohort 1, 9,593 DEGs1 were identified between BRCA and control samples, with 5,981 genes up-regulated and 3,612 genes down-regulated in BRCA samples (*p* < 0.05) (Fig. 2A and B). Similarly, in training cohort 2, 506 DEGs2 were identified between OB and control samples, which included 445 up-regulated genes and 61 down-regulated genes in OB samples (Fig. 2C and D). The up-regulated genes from DEGs1 and DEGs2 were intersected, yielding an intersecting gene set 1 comprised of 136 genes (Fig. 2E). Simultaneously, the down-regulated genes across the DEGs1 and DEGs2 were intersected to obtain an intersecting gene set 2 consisting of 42 genes (Fig. 2F). Upon merging both intersecting sets, a total of 178 intersecting genes were gained. Subsequently, the intersection of 178 intersecting genes, 410 key module genes 1, and 588 key module genes 2 yielded 27 candidate genes (Fig. 2G).

### Enrichment analysis of 27 candidate genes

GO annotation of the 27 candidates yielded 110 biological-process terms (e.g., hemostasis, blood coagulation, ossification; *p* < 0.05).(Supplementary Fig. 1A, Supplementary Table 2). Meanwhile, 10 cellular component (CC) entries were found, including “collagen-containing extracellular matrix”, “endoplasmic reticulum lumen”, and “basement membrane”, as well as 15 molecular function (MF) entries, encompassed “extracellular matrix binding”, “collagen binding”, and “peptidase regulator activity” (*p* < 0.05) (Supplementary Fig. 1A, Supplementary Table 2). Furthermore, 13 KEGG pathways were identified, like “ECM-receptor interaction”, “complement and coagulation cascades”, and “PI3K-Akt signaling pathway” (*p* < 0.05) (Supplementary Fig. 1B, Supplementary Table 2). Additionally, these candidate genes were represented in the PPI network (Supplementary Fig. 1C). Notably, F2R, SFRP2, and MXRA5 exhibited strong interactions with other genes, such as SERPINE1 and CDH11, highlighting the strong protein-level associations among them. These analyses provided a significant foundation for the functional significance of candidate genes in the progression of BRCA and OB.

### Selection of F2R and MXRA5 as biomarkers

**Fig. 3:**
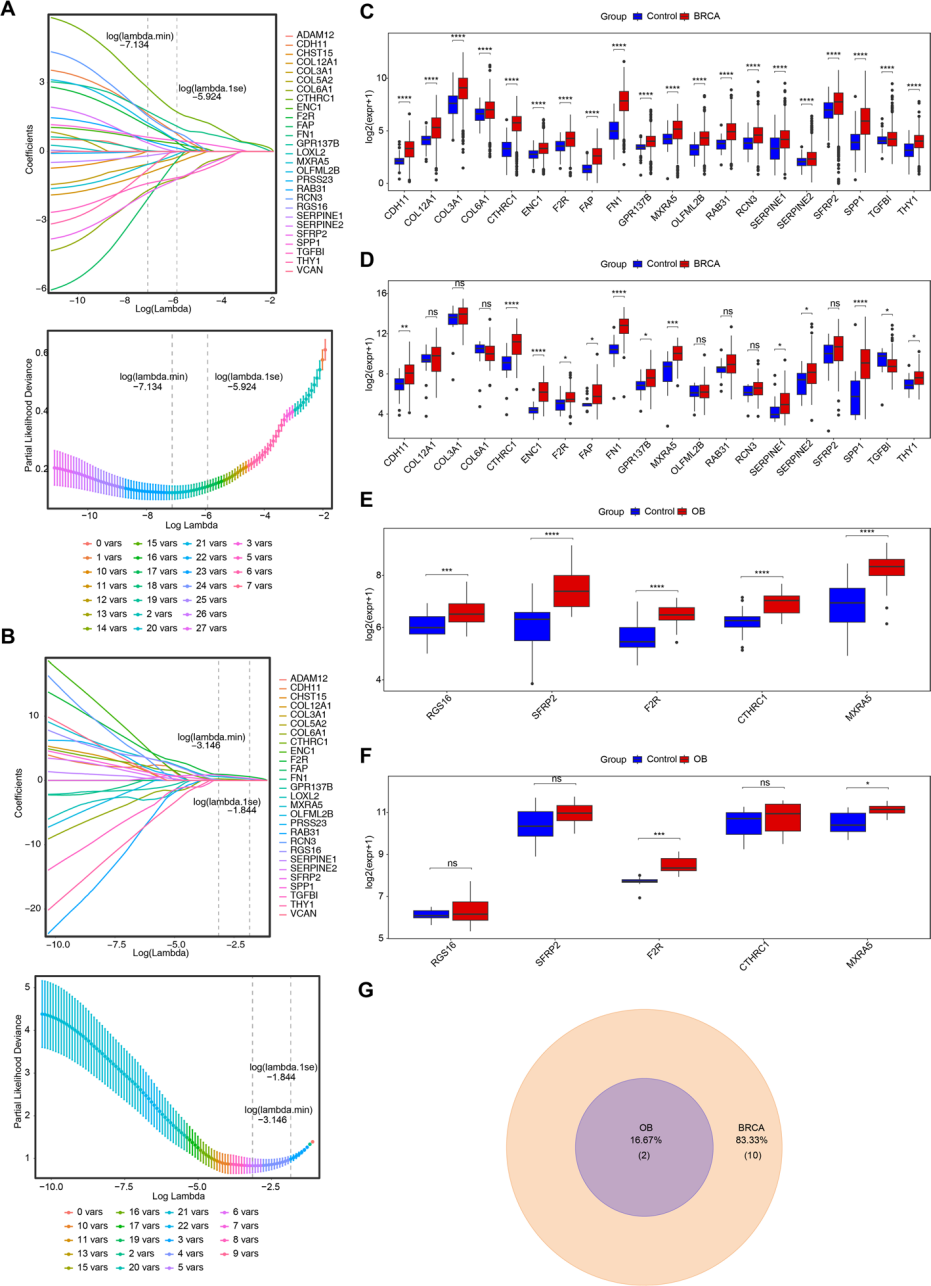
Comprehensive Analysis of Key Gene Identification and Validation. **A** LASSO coefficient path plot and LASSO cross-validation curve plot for Training Set 1; **B** LASSO coefficient path plot and LASSO cross-validation curve plot for Training Set 2; **C** Gene expression levels of signature genes in BRCA patient samples and control samples from Training Set 1; **D** Gene expression levels of signature genes in BRCA patient samples and control samples from Validation Set 1; **E** Gene expression levels of signature genes in OB patient samples and control samples from Training Set 2; **F** Gene expression levels of signature genes in OB patient samples and control samples from Validation Set 2; **G** Identification of Key Genes

Following this, the LASSO algorithm was applied to identify potential biomarkers in training cohorts 1 and 2, leading to the selection of 20 candidate biomarkers for BRCA (log(lambda.min) = -7.134) and 5 candidate biomarkers for OB (log(lambda.min) = -3.146) (Fig. 3A and B). The frequency distribution of gene selection across the analysis process and the coefficient trajectories are presented in Supplementary Fig. 2A, B.

Further analysis and expression validation were conducted to determine biomarkers. It was observed that in both training cohort 1 and validation cohort 1, 12 genes showed significant expression differences with consistent trends (*p* < 0.05) (Fig. 3C and D). Twelve genes (including CDH11, F2R, MXRA5, etc.) were consistently up-regulated in breast-cancer tissues (*p* < 0.05). Among them, F2R and MXRA5 were also elevated in obesity datasets (*p* < 0.05), qualifying as dual-disease biomarkers (Fig. 3E and F). Permutation testing (1,000 iterations) confirmed that the co-selection of F2R and MXRA5 was statistically significant across both the TCGA (*P* = 0.008) and OB (*P* = 0.020) cohorts. Detailed statistical metrics, including raw P-values and BH adjusted P-values for each cohort, are provided in Supplementary Table 3. These results substantiate the robust identification of these key biomarkers.

The subsequent intersection of biomarkers for BRCA and biomarkers for OB revealed 2 biomarkers associated with the YAP/TAZ pathway in both BRCA and OB (Fig. 3G), namely F2R and MXRA5. Coincidentally, within both training and validation cohorts for BRCA and OB, F2R and MXRA5 all presented AUC values greater than 0.7, further demonstrating their robust capacity to differentiate between BRCA and control samples, as well as between OB and control samples (Supplementary Fig. 3).

### Development of ANN models with robust predictive performance

**Fig. 4:**
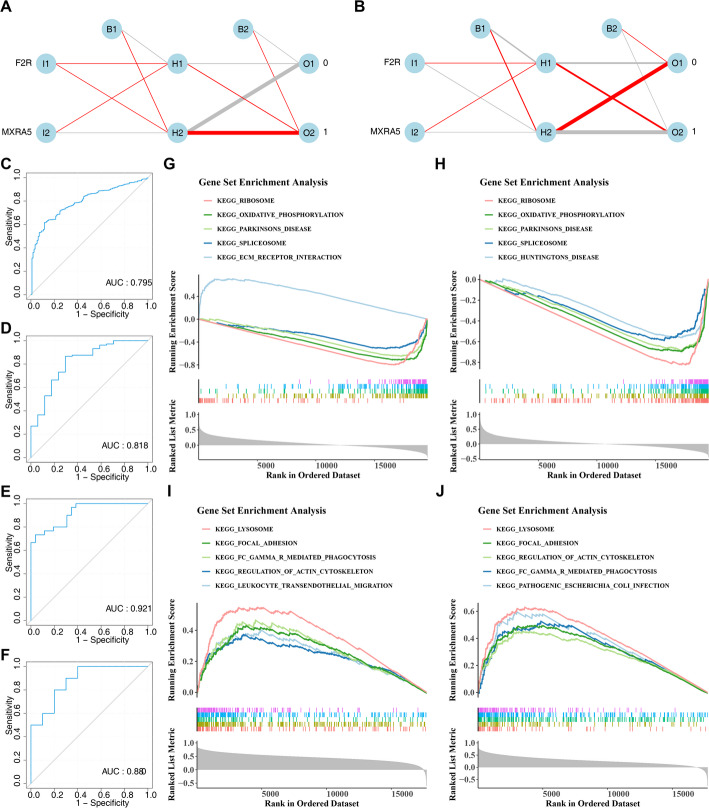
ANN model construction and functional enrichment. **A** Neural Network Topology for Training Set 1; **B** Neural Network Topology for Training Set 2; **C** ROC Curve of the ANN Model for Training Set 1; **D** ROC Curve of the ANN Model for Validation Set 1; **E** ROC Curve of the ANN Model for Training Set 2; **F** ROC Curve of the ANN Model for Validation Set 2; **G** GSEA Enrichment Results for Gene F2R in Training Set 1 (Top 5 Pathways); **H** GSEA Enrichment Results for Gene *MXRA5* in Training Set 1 (Top 5 Pathways); **I** GSEA Enrichment Results for Gene F2R in Training Set 2 (Top 5 Pathways); **J** GSEA Enrichment Results for Gene *MXRA5* in Training Set 2 (Top 5 Pathways)

Based on the above data, the ANN models were constructed in training cohorts 1 and 2 to maximally assess the risk of BRCA and OB, respectively (Fig. 4A and B). During model training, the loss (SSE) decreased rapidly in the initial iterations and subsequently plateaued, indicating convergence of the model (Supplementary Fig. 4A, B). The ROC curves underscored the credible predictive value of ANN models for BRCA and OB, with AUC values of 0.795 in training cohort 1 and 0.818 in validation cohort 1 for BRCA (Fig. 4C and D), and 0.921 in training cohort 2 and 0.880 in validation cohort 2 for OB (Fig. 4E and F). These results signified the ANN models robust ability to assess occurrence probability in BRCA and OB patients, respectively, though further validation in larger clinical samples was needed.

### Functional analysis of biomarkers

To explore the potential mechanisms of biomarkers, GSEA was conducted. In training cohort 1, F2R was significantly enriched in 76 pathways, while MXRA5 was enriched in 81 pathways (Supplementary Table 4). GSEA revealed shared enrichment of F2R and MXRA5 in ribosome, oxidative phosphorylation and spliceosome pathways (Fig. 4G and H); F2R additionally mapped to ECM-receptor interaction (Fig. 4G). These findings suggested that F2R and MXRA5 might influence the development of BRCA by affecting biological signaling pathways related to cell proliferation, energy metabolism, and RNA processing, which were crucial for tumor progression.

Furthermore, in training cohort 2, F2R was significantly enriched in 92 pathways, while MXRA5 was enriched in 74 pathways (Supplementary Table 4). F2R and MXRA5 were commonly enriched in pathways such as “regulation of actin cytoskeleton”, “lysosome”, “focal adhesion”, and “FC gamma R mediated phagocytosis” (Fig. 4I and J). Simultaneously, F2R was involved in the “leukocyte transendothelial migration” pathway (Fig. 4I), while MXRA5 participated in the “pathogenic escherichia coli infection” pathway (Fig. 4J). These pathways covered a wide range of biological processes, from immune response and cell adhesion to cytoskeletal dynamics and infection processes, demonstrating the complex pathways and regulatory mechanisms by which F2R and MXRA5 might influence OB.

Taken together, these findings underscored the critical roles of F2R and MXRA5 in regulating diverse biological functions that contributed to tumor progression, immune responses, and tissue remodeling, offering new insights into potential therapeutic targets for BRCA and OB.

### Revealing immune cell infiltration in BRCA and OB

Immune cell infiltration was a key process in the onset and progression of many diseases. By analyzing the distribution and infiltration proportions of each immune cell within diseased tissues, it was possible to reveal how these cells participated in the pathogenesis. Based on this, immune cell infiltration abundance was studied in 2 different cohorts.

**Fig. 5:**
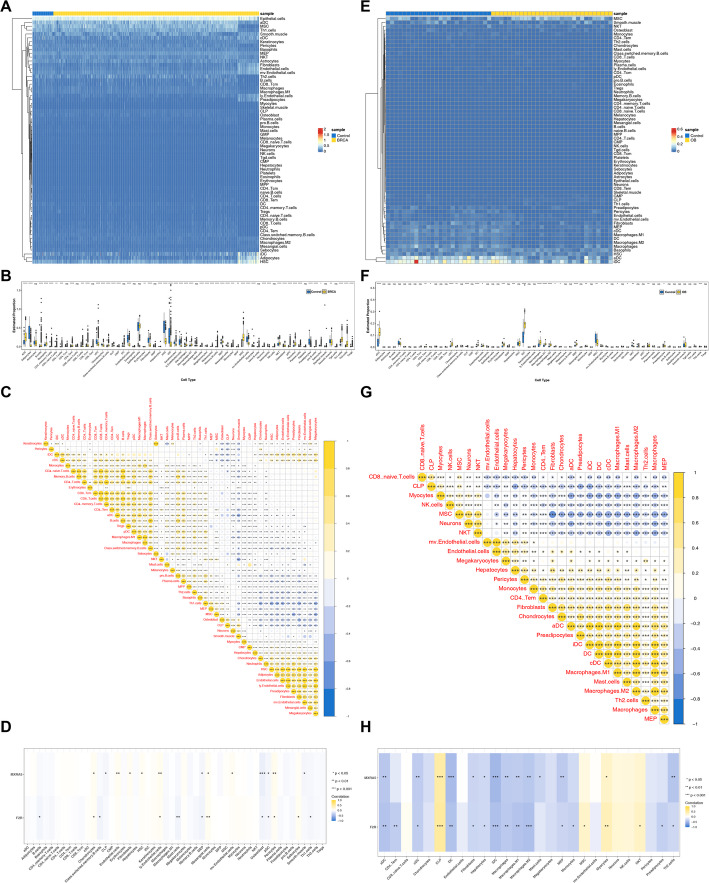
Comprehensive analysis of immune infiltration. **A** Heatmap of ssGSEA-Inferred Immune Cell Abundances in BRCA; **B** Differential Immune Cell Infiltration between BRCA and Controls; **C** Correlation Analysis Between Differential Immune Cells in BRCA; **D** Correlation Analysis Between Key Genes and Differential Immune Cells in BRCA; **E** Heatmap of ssGSEA-Inferred Immune Cell Abundances in OB; **F** Differential Immune Cell Infiltration between OB and Controls; **G** Correlation Analysis Between Differential Immune Cells in OB; **H** Correlation Analysis Between Key Genes and Differential Immune Cells in OB

In training cohort 1, the infiltration profiles of 64 immune infiltrating cells in the BRCA and control samples were visualized (Fig. 5A). Then, box plot further visualized these differences, identifying significant disparities in 50 types of immune infiltrating cells (*p* < 0.05) (Fig. 5B, Supplementary Table 5). Interestingly, most differential immune infiltrating cells exhibited significant correlations among themselves (|cor| > 0.30, *p* < 0.05), highlighting potential interactions and coordination within the immune microenvironment of BRCA patients (Fig. 5C, Supplementary Table 6). Meanwhile, biomarkers were found to correlate significantly with some differential immune cells (|cor| > 0.30, *p* < 0.05) (Fig. 5D, Supplementary Table 7). These findings suggested that modulating the expression of these genes or the associated immune infiltrating cells could provide therapeutic opportunities for BRCA.

Similarly, in training cohort 2, the infiltration profiles of 64 immune infiltrating cells in the OB and control samples were presented (Fig. 5E), revealing significant disparities in 27 types of immune infiltrating cells (*p* < 0.05) (Fig. 5F, Supplementary Table 5). Remarkably, the majority of the differential immune infiltrating cells displayed notable correlations with each other (|cor| > 0.30, *p* < 0.05) (Fig. 5G, Supplementary Table 6), and biomarkers were found to be significantly negatively correlated with most of these cell types (|cor| > 0.30, *p* < 0.05) (Fig. 5H, Supplementary Table 7). Modulating the expression of these genes or their associated immune infiltrating cells could be a promising strategy for intervening in OB.

Additionally, 23 types of immune infiltrating cells exhibited significant differences between BRCA and control samples, as well as between OB and control samples (Supplementary Table 5). These differences suggested distinct immune systems in both BRCA and OB, potentially offering insight into therapeutic targets.

To further evaluate the robustness of immune infiltration findings, CIBERSORT was applied as an independent immune deconvolution method. Using the LM22 signature matrix, 22 immune cell types were quantified. In the BRCA cohort, 14 immune cell types showed significant differences between disease and control groups. In the OB cohort, differential immune cell subsets were also identified. Notably, align with xCell analyses, most differentially infiltrated immune cells exhibited significant correlations with each other (|cor| > 0.30, *p* < 0.05), indicating coordinated immune alterations across samples (Supplementary Fig. 5).

### Evaluation of F2R and MXRA5 in immunomodulation: TIDE, IPS, and ESTIMATE algorithm analyses

**Fig. 6:**
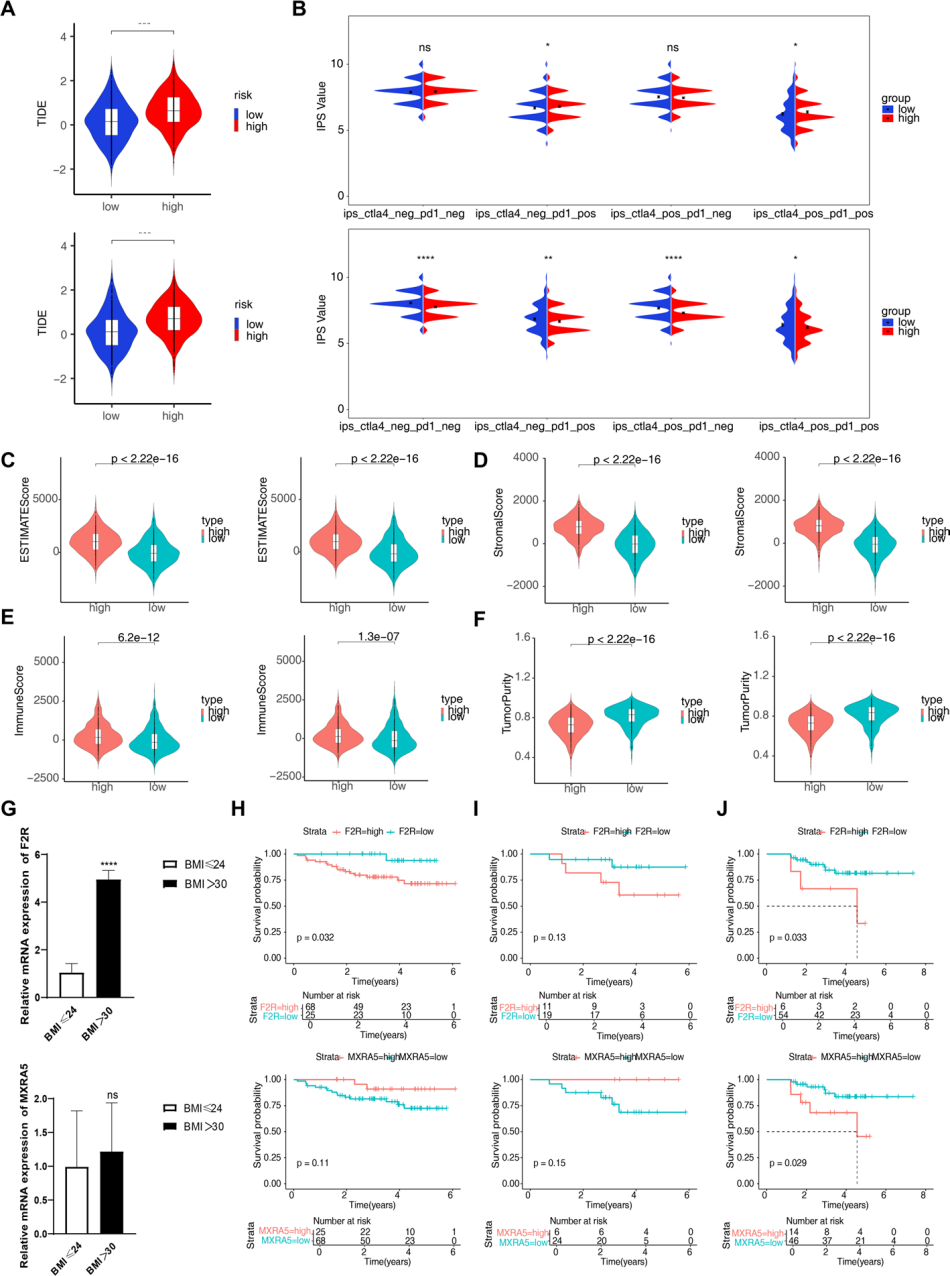
TIDE/IPS/ESTIMATE analysis, experimental validation and survival validation. **A** TIDE Analysis for F2R and MXRA5 in BRCA; **B** IPS Analysis for F2R and MXRA5 in BRCA; **C** ESTIMATE Score for F2R and MXRA5 in BRCA; **D** Stromal Score for F2R and MXRA5 in BRCA; **E** Immune Score for F2R and MXRA5 in BRCA; **F** Tumor Purity Analysis for F2R and MXRA5 in BRCA; **G** Relative mRNA expression of F2R and MXRA5 in breast adipose tissue from normal-weight and obese patients with benign breast disease; **H** Survival Analysis for F2R and MXRA5 in Luminal A subtype; **I** Survival Analysis for F2R and MXRA5 in Luminal B subtype; **J** Survival Analysis for F2R and MXRA5 in TNBC subtype;

High F2R or MXRA5 expression was associated with higher TIDE scores (*p* < 0.001), indicating a propensity for immune evasion, potentially through shaping an immunosuppressive tumor microenvironment (Fig. 6A). F2R low-expression was associated with significantly reduced IPS values, suggesting its potential role in promoting an immunosuppressive microenvironment via regulation of immune checkpoints like CTLA-4 and PD-1. Conversely, low MXRA5 expression correlated with higher IPS across multiple subtypes, indicating a possible enhanced sensitivity to anti-CTLA-4/PD-1 immunotherapy. These results imply that F2R may facilitate immune escape, whereas MXRA5 may serve as a negative predictor of immunotherapy response (Fig. 6B). The high-expression groups of both F2R and MXRA5 genes exhibited significantly elevated StromalScore, ImmuneScore, and ESTIMATEScore compared to their low-expression counterparts (*p* < 0.001). Conversely, tumor purity was markedly lower in the high-expression groups (*p* < 0.001) (Fig. 6C, D, E and F). It should be noted that the TIDE algorithm is inherently designed for high-dimensional, genome-wide expression data. In this study, the analysis based on the expression of only two biomarkers, F2R and MXRA5, represents an exploratory application of TIDE, and its findings would require further validation through genome-wide data or multi-gene joint analysis.

### Unraveling regulatory networks of biomarkers

The multiMiR (v 1.16.0) package was employed to predict miRNAs that related to the biomarkers, identifying 28 miRNAs for F2R and 38 miRNAs for MXRA5 (Supplementary Table 8). Subsequently, a total of 120 TFs predicted for F2R and 44 TFs associated with MXRA5 were identified (Supplementary Table 9). The TF-mRNA-miRNA regulatory network was developed, including 63 miRNAs such as hsa-miR-590-3p, hsa-miR-335-3p, and hsa-miR-1277-5p, along with 9 TFs (the top 5 TFs for each biomarker) like SPI1, FOXA1, and ESR1 (Supplementary Fig. 6A). These results facilitated further exploration of the role of these interactions in mechanisms of BRCA and OB.

### Dissection of drug sensitivity in BRCA and targeted drugs in OB

The drug sensitivity analysis results revealed that the top 5 chemotherapy drugs were AZD8055, BMS.754,807, Cytarabine, MS.275, and Vorinostat, with computationally predicted associations observed between the biomarkers and most drugs (Supplementary Fig. 6B, Supplementary Table 10). Specifically, F2R demonstrated significant negative correlations with AZD8055 (cor = -0.34, *p* < 0.001), BMS.754,807 (cor = -0.96, *p* < 0.001), and Cytarabine (cor = -0.93, *p* < 0.001), while showing significant positive correlation with Vorinostat (cor = 0.31, *p* < 0.001). On the other hand, MXRA5 showed significant positive correlations with AZD8055 (cor = 0.47, *p* < 0.001), MS.275 (cor = 0.67, *p* < 0.001), and Vorinostat (cor = 0.92, *p* < 0.001), while it exhibited significant negative correlations with BMS.754,807 (cor = -0.81, *p* < 0.001) and Cytarabine (cor = -0.33, *p* < 0.001). These results suggested that F2R and MXRA5 might serve as potential targets for predicting chemotherapy drug sensitivity and guiding personalized treatment strategies.

Subsequently, using DSigDB, potential targeted drugs related to biomarkers were predicted for OB, resulting in 34 potential drugs identified for F2R and 8 potential drugs for MXRA5 (Supplementary Table 11). Based on this information, a drug-biomarkers network was constructed (Supplementary Fig. 6C). Remarkably, 3 drugs were commonly predicted to target both biomarkers: progesterone, trichostatin A, and Valproic_Acid, suggesting their potential for dual-target therapy in the treatment of OB.

### Verification of biomarkers expression

Analysis of the relative mRNA expression levels revealed distinct patterns for F2R and MXRA5 across obesity (OB) and BRCA cohorts. BMI ≥ 30 showed markedly higher F2R levels than lean controls (*p* < 0.0001); MXRA5 displayed only a numerical increase (Fig. 6G). In BRCA tissues, both F2R (range: 0–40 arbitrary units) and MXRA5 (range: 0–25 arbitrary units) showed numerical increases relative to adjacent normal tissues; however, these differences lacked statistical significance (ns, *p* > 0.05 for both) (Supplementary Fig. 7A).

### Prognostic value of F2R and MXRA5 across breast cancer subtypes

In the Luminal A subtype, survival analysis of the F2R gene revealed a statistically significant difference in survival probability between the two groups (*p* = 0.032), whereas the difference observed for the MXRA5 gene was not statistically significant (*p* = 0.11) (Fig. 6H). In the Luminal B subtype, the survival differences for both the F2R and MXRA5 genes did not reach statistical significance (*p* = 0.13 and *p* = 0.15, respectively) (Fig. 6I). Conversely, among triple-negative breast cancers, high F2R or MXRA5 expression was associated with poorer survival (*p* = 0.033 and *p* = 0.029, respectively) (Fig. 6J).

## Discussion

By integrating transcriptomic profiles, WGCNA and machine-learning pipelines, we newly identify F2R and MXRA5 as candidate YAP/TAZ-associated genes shared between breast cancer and obesity at the transcriptomic level. Their consistent up-regulation across discovery and validation cohorts, together with ANN-based AUCs > 0.7, highlights translational potential for risk stratification.

The F2R gene encodes protease-activated receptor 1 (PAR1), a 425-amino-acid G protein-coupled receptor (GPCR) with seven transmembrane domains. Its unique activation involves proteolytic cleavage of the N-terminus (e.g., by thrombin) to expose the tethered ligand SFLLRN, triggering G protein signaling [37] that regulates tumor-associated processes like metastasis and angiogenesis [38]. Recent studies highlight PAR1’s role in remodeling tumor microenvironments: it promotes extracellular matrix remodeling in pancreatic cancer [39], and its antagonist SCH79797 synergizes with paclitaxel to suppress peritoneal metastasis in gastric cancer [40]. PAR1 also drives metabolic-associated steatotic liver disease (MASLD) in obese mice via nonhepatocellular cleavage and its selective modulation confers metabolic benefits in experimental obesity [41]. MXRA5, a matrix remodeling protein, has previously been reported as a oncogene in pancreatic cancer progression via PI3K-Akt-mTOR/EMT [42] and as a key lipid metabolism regulator by modulating PPARγ/CEBPα expression during preadipocyte differentiation and lipid droplet formation, linking its function to bariatric surgery-induced immunometabolic remodeling in subcutaneous adipose tissue [43].

Functional enrichment analysis further elucidated context-dependent pathways. In BRCA, F2R and MXRA5 were enriched in ribosome and oxidative phosphorylation pathways, which are critical for sustaining tumor process and energy demands [44, 45]. Conversely, in OB, these genes converged on lysosomal and focal adhesion pathways. Lysosomal dysfunction in adipose and liver drives obesity-related metabolic disorders by impairing protease activity and promoting inflammation/senescence [46]. Focal adhesion kinase sustains adipocyte survival during metabolic stress, enabling adipose expansion and insulin sensitivity [47]. The shared enrichment of the “energy and metabolic” pathway in both diseases suggests that YAP/TAZ may mediate the interplay between BRCA and obesity by regulating tumor metabolic flexibility and adipose stress adaptation, thereby mechanistically coupling metabolic perturbations to malignant progression.

Immune profiling showed a significant differences in the infiltration levels of M0, M1 macrophages both in breast cancer cohorts and obesity-associated groups. Further analysis revealed F2R and MXRA5 negatively correlated with all three macrophage(M0, M1, M2)subtypes in OB, suggesting their potential role as negative regulators of macrophage infiltration in obesity associated breast cancer. Moreover, In the OB group, immune profiling revealed a decrease in dendritic cell (DC) abundance, a feature that co-occurred with elevated expression of F2R and MXRA5. These genes correlated inversely with DC abundance, potentially impairing antigen presentation and T cell priming through dysregulated chemokine signaling and co-stimulatory pathways [48]. High F2R/MXRA5 expression is linked to elevated TIDE scores (immune escape), reduced IPS (impaired antigen presentation), and lower tumor purity (increased stromal/immune infiltration), aligning with the earlier observation that these genes correlate negatively with dendritic cells and macrophages—together indicating an immunosuppressive, T-cell-excluded microenvironment. Consistent with the immunosuppressive phenotype described above, elevated expression of F2R and MXRA5 was significantly associated with intrinsic resistance to the HDAC inhibitor Vorinostat. As Vorinostat is known to enhance anti-tumor immunity by blocking HDAC-mediated immunosuppression [49, 50]. our computational analysis revealing a positive correlation between F2R/MXRA5 expression and Vorinostat IC₅₀ values suggests these genes may be involved in intrinsic HDACi resistance. Taken together, these findings indicate that F2R and MXRA5 are not only correlated with reduced macrophage/dendritic cell infiltration and enhanced immune evasion but may also help identify tumors less likely to respond to Vorinostat monotherapy. This preliminary evidence warrants further investigation into whether they could serve as dual biomarkers to guide the rational selection of tumors that might benefit from combination epigenetic-immunotherapy strategies.

The lack of significant differential expression of F2R and MXRA5 between BRCA and adjacent tissues could be attributed to cohort heterogeneity, where the inclusion of diverse tumor subtypes and stages may have obscured subtype-specific effects. F2R’s marked upregulation in obesity underscores its role in metabolic-inflammatory crosstalk, potentially linked to YAP/TAZ-driven pathways [6]. The prognostic validation of F2R and MXRA5 highlighted their clinical relevance. Consistent with their proposed roles, elevated expression of both genes was significantly associated with poorer survival outcomes, notably within the TNBC subtype, a clinical context associated with high aggressiveness. This aligns with the known role of YAP/TAZ signaling in driving tumorigenesis in TNBC, a subtype devoid of targeted therapeutic options [51, 52]. The lack of significance in Luminal subtypes may reflect the dominance of estrogen receptor signaling pathways, potentially overshadowing the contribution of YAP/TAZ. The potent prognostic power in TNBC positions F2R and MXRA5 as valuable risk-stratification markers for this challenging patient cohort, potentially guiding more aggressive or novel therapeutic strategies upfront.

This study has several limitations. First, the training data used to identify obesity-related genes were derived from subcutaneous adipose tissue (SAT), which may not fully capture the biology of the local mammary microenvironment. This may partly explain why F2R and MXRA5 did not show more pronounced differential expression in breast cancer tissues. Future studies should directly validate their expression and function within mammary adipose tissue. Second, as a retrospective, cross-sectional analysis, this study identifies associations rather than causality between obesity and molecular changes. The inability to fully adjust for confounders like age and treatment history remains a limitation. To confirm the prognostic independence of F2R and MXRA5, future research should utilize prospective cohorts and rigorous multivariate modeling to account for a broader range of clinical covariates. Their prognostic independence should be further evaluated using multivariate analysis in prospective cohorts with comprehensive clinical annotation. Furthermore, the experimental validation was conducted in a relatively small sample size, which may limit the representativeness and robustness of the findings. Additionally, the lack of patient body mass index (BMI) data precluded a systematic assessment of the correlation between F2R/MXRA5 protein expression and obesity metrics. Future work should employ techniques such as immunohistochemistry in prospectively collected cohorts annotated with metabolic parameters to confirm protein-level expression and analyze its association with obesity features and patient outcomes, thereby strengthening the clinical relevance of these genes as potential obesity-driven biomarkers in breast cancer.

## Conclusion

This study identified F2R and MXRA5 as candidate YAP/TAZ-associated genes that may link obesity and breast cancer. Their expression levels correlated with features of immune suppression and metabolic reprogramming. More specifically, elevated expression of these genes was associated with an immunosuppressive tumor microenvironment characterized by reduced dendritic cell and macrophage infiltration and an increased potential for immune escape. Furthermore, computational analyses suggested a possible association between high F2R/MXRA5 expression and resistance to Vorinostat. Their prognostic associations in TNBC may provide preliminary evidence for risk stratification and hypothesis generation for future mechanistic and therapeutic studies in obesity-associated breast cancer.

## Supplementary Information

Supplementary Material 1.

## Data Availability

All datasets analyzed in this study are publicly available: BRCA data from TCGA ( [https://portal.gdc.cancer.gov](https:/portal.gdc.cancer.gov) ) and GEO (GSE42568, GSE25065), obesity data from GEO (GSE25401, GSE151839), and YAP/TAZ genes from MSigDB. Supplementary results are included. Analysis code is available from the corresponding author upon reasonable request.
