# Supplementary material for: F2R and MXRA5: the metabolic obesity-derived biomarkers for immunosuppression and poor survival in triple-negative breast cancer
Source: Discov Oncol. 2026 Apr 12;17:782. doi: 10.1007/s12672-026-04963-9 (PMC13201692; doi:10.1007/s12672-026-04963-9)
Supplement: Supplementary file 1 — Supplementary Material 1. [file 12672_2026_4963_MOESM1_ESM.zip › 12672_2026_4963_MOESM1_ESM/Figure S5.pdf]

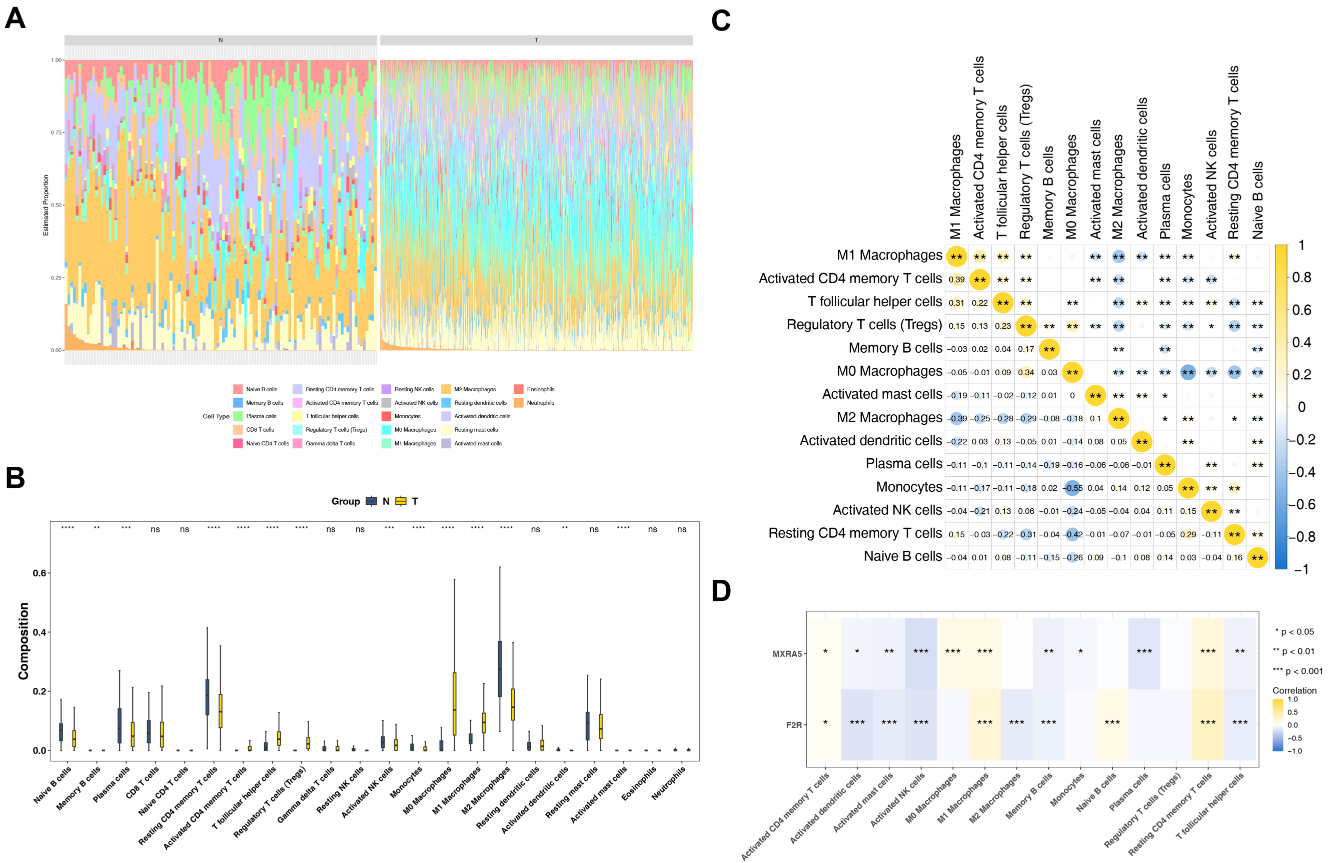
A
N
T
Estimated Proportion
1.00
0.75
0.50
0.25
0.00
Cell Type
Naive B cells
Memory B cells
Plasma cells
CD8 T cells
Naive CD4 T cells
Resting CD4 memory T cells
Activated CD4 memory T cells
T follicular helper cells
Regulatory T cells (Tregs)
Gamma delta T cells
Resting NK cells
Activated NK cells
Monocytes
M0 Macrophages
M1 Macrophages
M2 Macrophages
Resting dendritic cells
Activated dendritic cells
Resting mast cells
Activated mast cells
Eosinophils
Neutrophils
B
Group N T
Composition
0.0
0.2
0.4
0.6
**** ** *** ns ns **** **** **** **** ns ns *** **** **** **** **** ns ** ns **** ns ns
Naive B cells
Memory B cells
Plasma cells
CD8 T cells
Naive CD4 T cells
Resting CD4 memory T cells
Activated CD4 memory T cells
T follicular helper cells
Regulatory T cells (Tregs)
Gamma delta T cells
Resting NK cells
Activated NK cells
Monocytes
M0 Macrophages
M1 Macrophages
M2 Macrophages
Resting dendritic cells
Activated dendritic cells
Resting mast cells
Activated mast cells
Eosinophils
Neutrophils
C
M1 Macrophages
Activated CD4 memory T cells 0.39
T follicular helper cells 0.31 0.22
Regulatory T cells (Tregs) 0.15 0.13 0.23
Memory B cells −0.03 0.02 0.04 0.17
M0 Macrophages −0.05 −0.01 0.09 0.34 0.03
Activated mast cells −0.19 −0.11 −0.02 −0.12 0.01 0
M2 Macrophages −0.39 −0.25 −0.28 −0.29 −0.08 −0.18 0.1
Activated dendritic cells −0.22 0.03 0.13 −0.05 0.01 −0.14 0.08 0.05
Plasma cells −0.11 −0.1 −0.11 −0.14 −0.19 −0.16 −0.06 −0.06 −0.01
Monocytes −0.11 −0.17 −0.11 −0.18 0.02 −0.55 0.04 0.14 0.12 0.05
Activated NK cells −0.04 −0.21 0.13 0.06 −0.01 −0.24 −0.05 −0.04 0.04 0.11 0.15
Resting CD4 memory T cells 0.15 −0.03 −0.22 −0.31 −0.04 −0.42 −0.01 −0.07 −0.01 −0.05 0.29 −0.11
Naive B cells −0.04 0.01 0.08 −0.11 −0.15 −0.26 0.09 −0.1 0.08 0.14 0.03 −0.04 0.16
1
0.8
0.6
0.4
0.2
0
−0.2
−0.4
−0.6
−0.8
−1
D
MXRA5
F2R
* p < 0.05
** p < 0.01
*** p < 0.001
Correlation
1.0
0.5
0.0
−0.5
−1.0
Activated CD4 memory T cells
Activated dendritic cells
Activated mast cells
Activated NK cells
M0 Macrophages
M1 Macrophages
M2 Macrophages
Memory B cells
Monocytes
Naive B cells
Plasma cells
Regulatory T cells (Tregs)
Resting CD4 memory T cells
T follicular helper cells
