# Supplementary figures and images for: F2R and MXRA5: the metabolic obesity-derived biomarkers for immunosuppression and poor survival in triple-negative breast cancer
Source: Discov Oncol. 2026 Apr 12;17:782. doi: 10.1007/s12672-026-04963-9 (PMC13201692; doi:10.1007/s12672-026-04963-9)

A

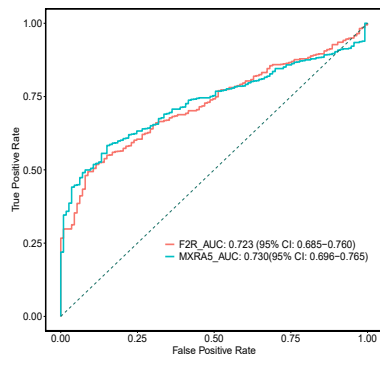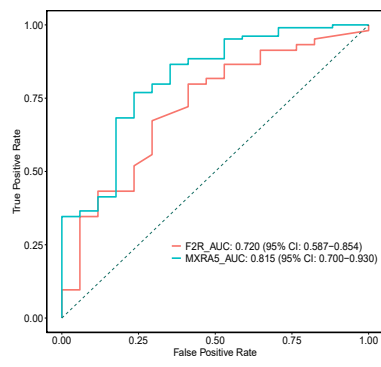

B

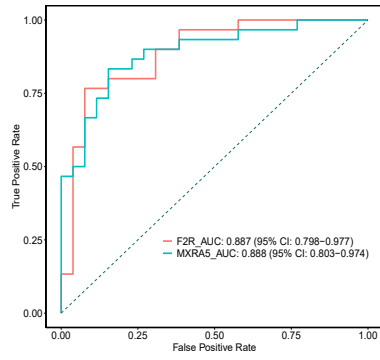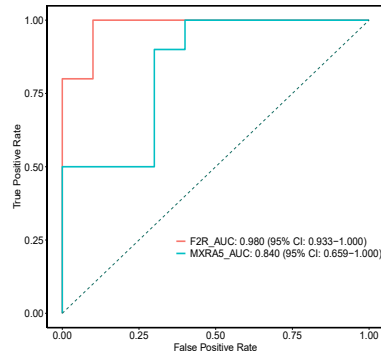

Supplement: Supplementary file 1 — Supplementary Material 1. [file 12672_2026_4963_MOESM1_ESM.zip › 12672_2026_4963_MOESM1_ESM/Figure S3.pdf]

A

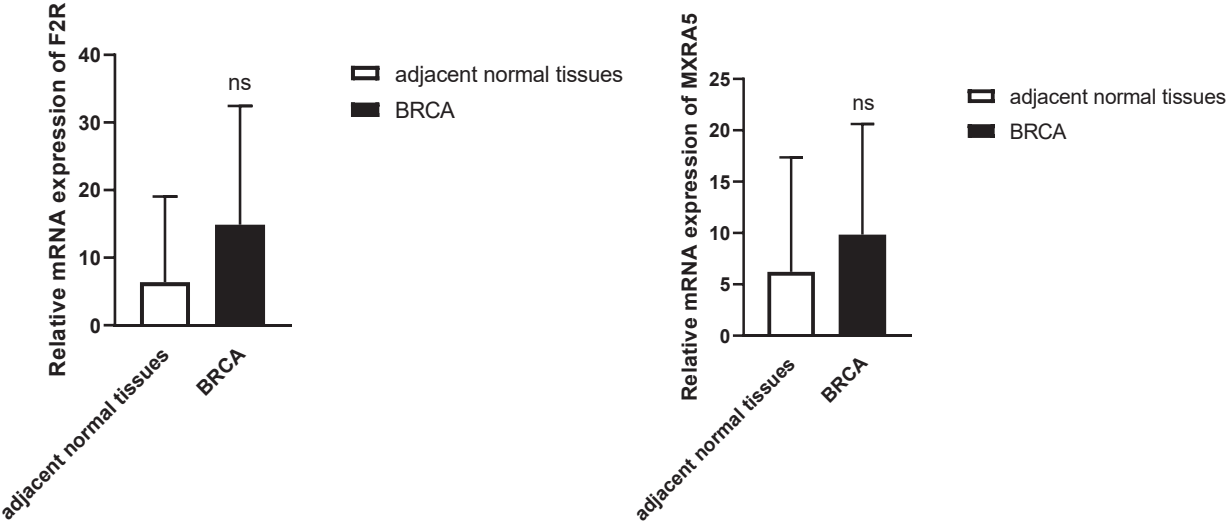

Supplement: Supplementary file 1 — Supplementary Material 1. [file 12672_2026_4963_MOESM1_ESM.zip › 12672_2026_4963_MOESM1_ESM/Figure S7.pdf]

A

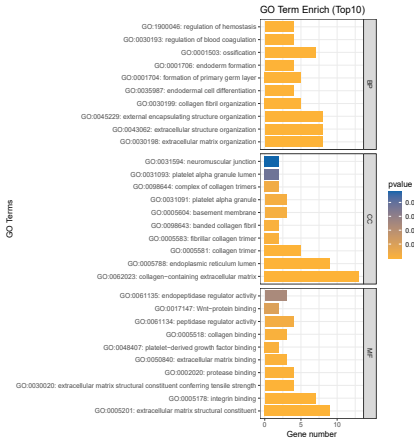

B

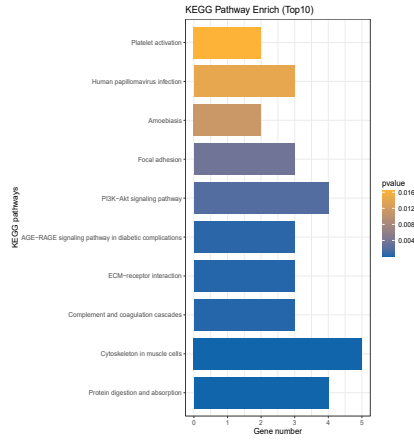

C

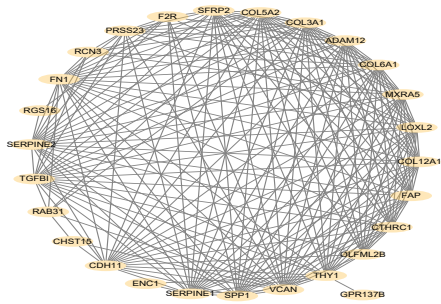

Supplement: Supplementary file 1 — Supplementary Material 1. [file 12672_2026_4963_MOESM1_ESM.zip › 12672_2026_4963_MOESM1_ESM/Figure S1.pdf]

**A**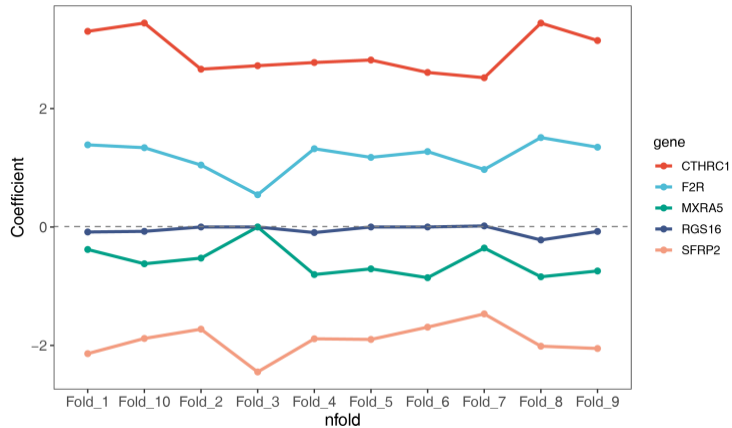**B**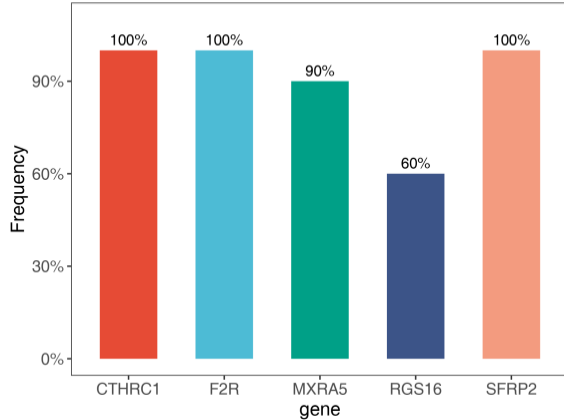

Supplement: Supplementary file 1 — Supplementary Material 1. [file 12672_2026_4963_MOESM1_ESM.zip › 12672_2026_4963_MOESM1_ESM/Figure S2.pdf]

**A****BP Neural Network Training Loss Curve (TCGA)**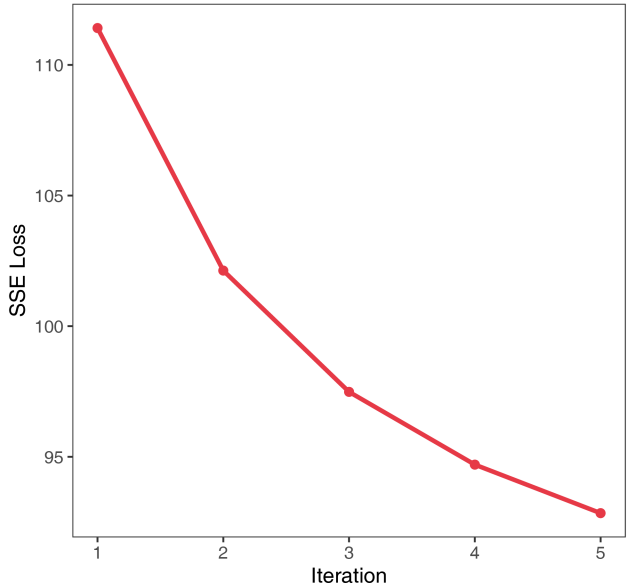**B****BP Neural Network Training Loss Curve (Obese)**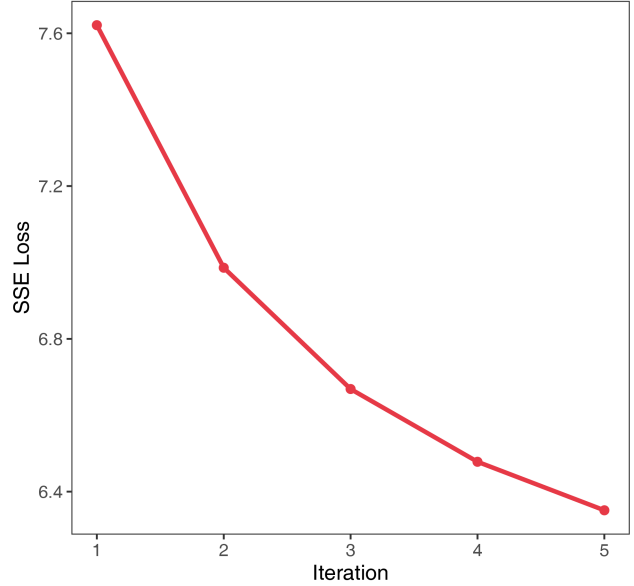

Supplement: Supplementary file 1 — Supplementary Material 1. [file 12672_2026_4963_MOESM1_ESM.zip › 12672_2026_4963_MOESM1_ESM/Figure S4.pdf]

B

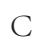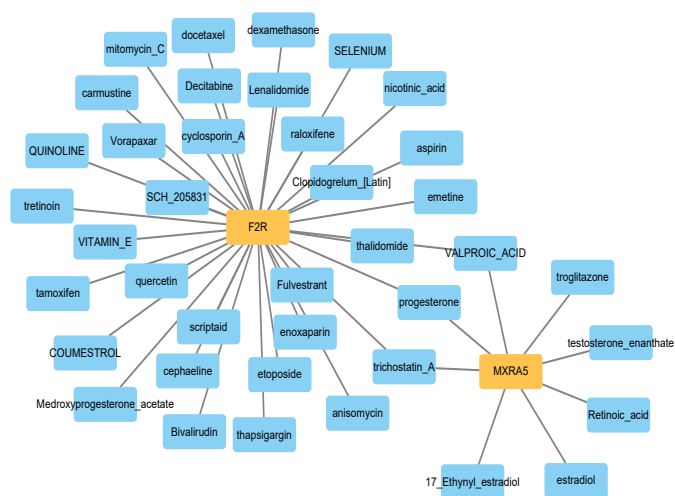

Supplement: Supplementary file 1 — Supplementary Material 1. [file 12672_2026_4963_MOESM1_ESM.zip › 12672_2026_4963_MOESM1_ESM/Figure S6.pdf]
